# The “safe-line” technique as theoretical additional attempt to mitigate spinal cord ischemia after urgent complete endovascular exclusion of a thoracoabdominal aortic aneurysm

**DOI:** 10.1016/j.jvscit.2023.101215

**Published:** 2023-05-16

**Authors:** Michele Piazza, Francesco Squizzato, Marco James Bilato, Edoardo Forcella, Franco Grego, Michele Antonello

**Affiliations:** Vascular and Endovascular Surgery Division, Department of Cardiac, Thoracic, Vascular Sciences, and Public Health, School of Medicine, Padua University, Padua, Italy

**Keywords:** Aortic aneurysm, Endovascular aneurysm repair, Spinal cord ischemia, Thoracoabdominal, Paraplegia

## Abstract

We describe the feasibility of a technique for temporary aneurysm sac reperfusion after endovascular single-stage thoracoabdominal aortic aneurysm exclusion, to be used in the case of postoperative spinal cord ischemia. Two cases were treated for impending rupture of a thoracoabdominal aortic aneurysm. Before completion of sac exclusion, a supplementary buddy wire (V-18 control guidewire; Boston Scientific) was advanced in parallel fashion from the left percutaneous femoral access into the aneurysmal sac on the posterior aspect of the endograft. Distal aneurysm exclusion was completed using the main superstiff guidewire, and the femoral access was closed with a percutaneous closure device (ProGlide; Abbott) in standard fashion, leaving in place the sole V-18 guidewire, draped in sterile fashion. In the case of spinal cord ischemia, the “safe-line” can be rapidly used for spinal reperfusion after trans-sealing exchange with a 6F, 65-cm-long Destination sheath (Terumo) connected to a 6F introducer on the contralateral femoral artery.

Endovascular repair of thoracoabdominal aortic aneurysms (TAAAs) represents a valid treatment option, owing to its low 30-day mortality and complication rates.^1^^,^^2^ Although endovascular treatment has evolved greatly, spinal cord ischemia (SCI) and consequent paraplegia or paraparesis continues to be a devastating complication for the patient, family, and surgeon. Large experience from different centers report a SCI rate ranging from 4% for multistage to ≤18% for single-stage endovascular repair.^3^^,^^4^

SCI genesis is multifactorial, with arterial embolization, coverage of long aortic segments, hypotension, anemia, and/or insufficient collateral vessels the main predisposing factors. Over the years, several SCI preventive strategies have been developed. The most common consists of staging the procedure to allow for gradual spinal cord adaptation before sac exclusion. This can be obtained by two-step aortic coverage, temporary aneurysm sac perfusion (TASP), or minimally invasive segmental artery coil embolization. However, these approaches lack the possibility for allowing for spinal cord reperfusion in the case of SCI occurring after aneurysm exclusion.^5^

In the present report, we aim to demonstrate the technical feasibility of maintaining a temporary guidewire into the aneurysm sac to be used as a rapid route for fast reperfusion in the case of SCI occurring ≤48 hours after complete endovascular TAAA exclusion.

## Technique

**Fig 1:**
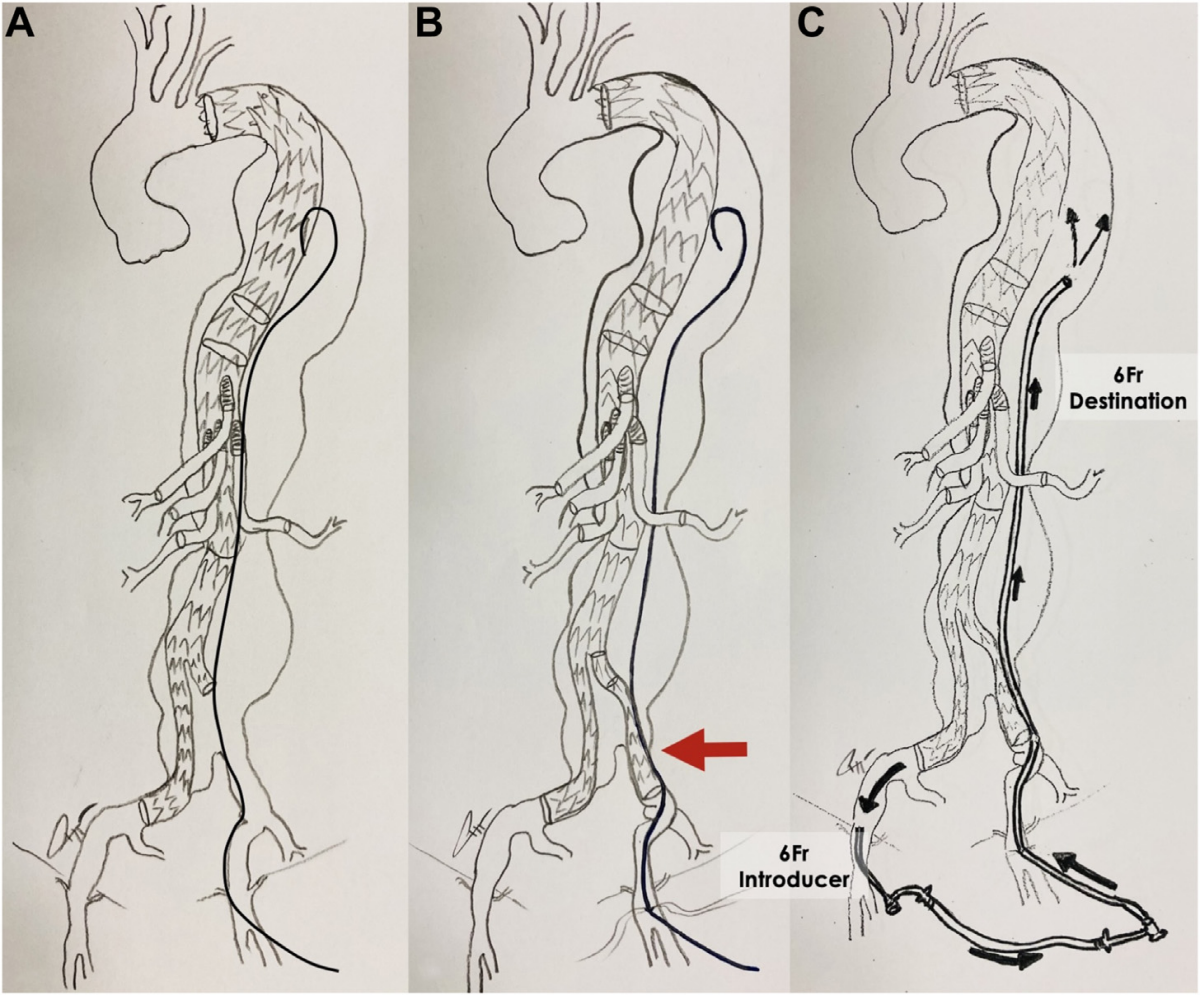
Schematic drawings representing the “safe-line technique.” **A,** From left femoral artery access, before deployment of the distal aortic graft component, a 0.018-in. guidewire is advanced into the aneurysmal sac. **B,** Endovascular aneurysm exclusion completed via deployment of the distal aorto-bifurcated endograft from right femoral artery access, with the prepositioned 0.018-in. guidewire left in place. **C,** If postoperative spinal cord ischemia (SCI) occurs, the “safe line” is used to advance into the aneurysm sac, a 6F Destination sheath, which is then connected to the contralateral femoral artery access, allowing for quick sac reperfusion.

The technique is based on leaving a 0.018-in. guidewire (a “safe line”) into the aneurysmal sac from femoral access. In the case of SCI occurring within 48 hours after complete exclusion, this guidewire can be rapidly exchanged for a 6F long sheath, which is advanced into the sac via a trans-sealing route and connected to contralateral femoral artery access for rapid sac reperfusion (Fig 1 and Supplementary Video, online only).

We report two cases of symptomatic impending TAAA rupture treated endovascularly in an urgent setting with long-segment aortic coverage and a single-stage procedure. The patients provided written informed consent for the report of their case details and imaging studies.

## Case report

### Patient 1

**Fig 2:**
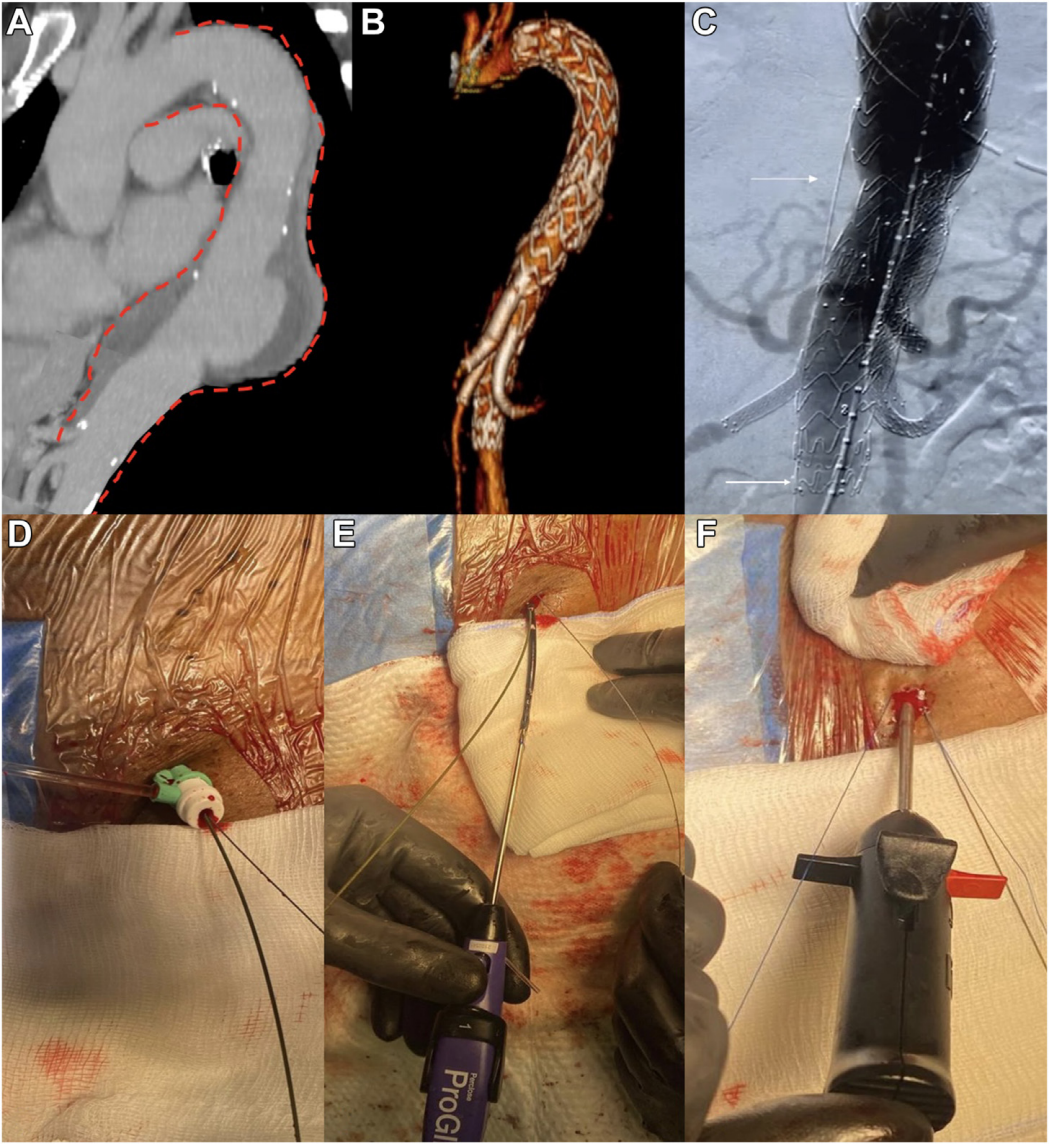
**A,** Computed tomography angiogram showing a thoracoabdominal aortic aneurysm (TAAA) with associated intramural hematoma. **B,** Postoperative computed tomography angiogram showing successful endovascular treatment with branched endovascular repair. **C,** Completion angiogram showing correct positioning of the endografts and bridging stents, with the V-18 guidewire (*white arrow*) left in the aneurysm sac, entering from the distal edge of the endograft. **D-F,** Percutaneous closure of left femoral artery access, with the V-18 guidewire left in place.

A 65-year-old woman with a symptomatic type I TAAA and associated intramural hematoma (Fig 2, *A*) was treated by proximal thoracic endografting and deployment of an off-the-shelf inner branch device (E-nside; Artivion) from the right femoral artery access, landing proximally in zone 3 and distally in the middle infrarenal abdominal aorta (Fig 2, *B*).^6^

Before deployment of the distal component, a 0.018-in. guidewire (V-18 control guidewire; Boston Scientific) was advanced from the left side over a 6F introducer into the aneurysmal sac and left behind the main inner branched graft. Once the procedure was completed, the V-18 guidewire was left in position (Fig 2, *C*), the 6F introducer was removed, and the access site was closed using a percutaneous closure device (Perclose ProGlide; Abbott; Fig 2, *D-F*).

### Patient 2

**Fig 3:**
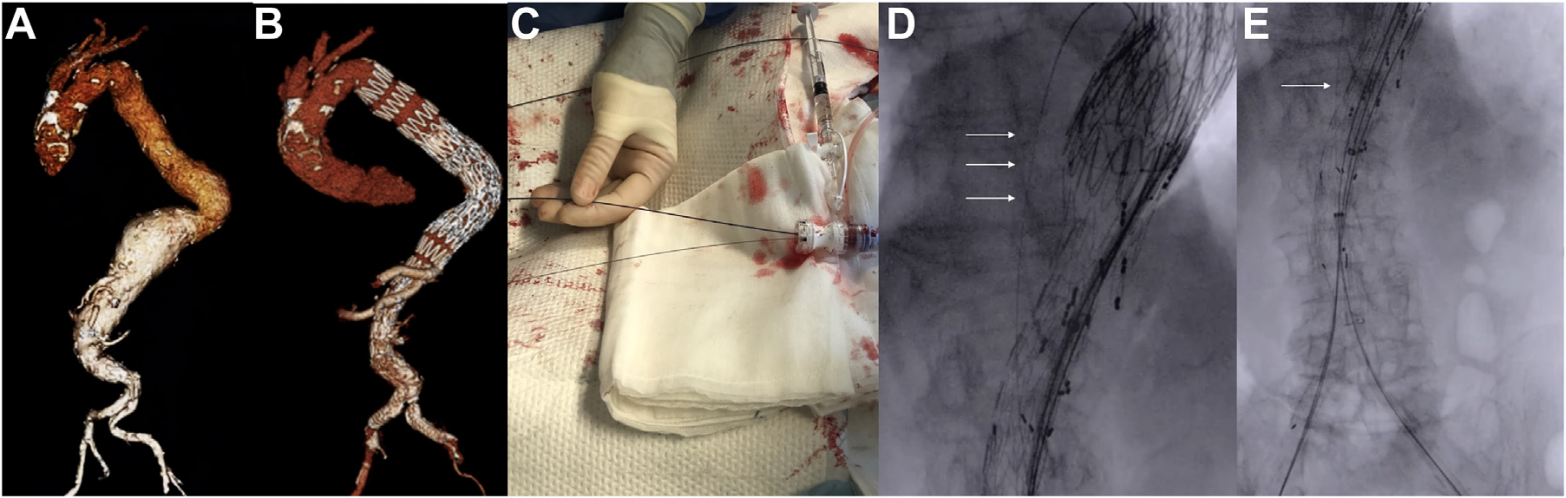
**A,** Computed tomography angiogram showing a thoracoabdominal aortic aneurysm (TAAA). **B,** Postoperative computed tomography angiogram showing successful TAAA treatment. **C,** Detail of left femoral artery access: a DrySeal introducer sheath is used to accommodate both the 0.035-in. superstiff guidewire used for deployment of the left limb and the V-18 guidewire used as a “safe line.” **D** and **E,** Intraoperative fluoroscopy showing the guidewire properly positioned behind the aortic main graft (*arrows*).

A 76-year-old woman presented with a 5-day history of abdominal and back pain and a type III TAAA (Fig 3, *A*). From the right femoral artery access, a proximal thoracic endograft, off-the-shelf outer branch device (T-branch; Cook Medical), and distal endovascular aneurysm repair graft were deployed, landing proximally in zone 3 and distally in both common iliac arteries (Fig 3, *B*).

A 16F DrySeal sheath was inserted into the left femoral artery and used to accommodate a superstiff Amplatz guidewire (Boston Scientific) and V-18 guidewire, which were advanced in parallel fashion into the aneurysmal sac and left behind the main graft (Fig 3, *C* and *D*). From this same introducer sheath, the left iliac limb was deployed over the superstiff guidewire (Fig 3, *E*).

Once the procedure was completed, the Amplatz guidewire was removed, and the V-18 guidewire was left in position. The 16F introducer was removed, and the access site was closed using two prepositioned ProGlide systems (Perclose ProGlide; Abbott).

**Fig 4:**
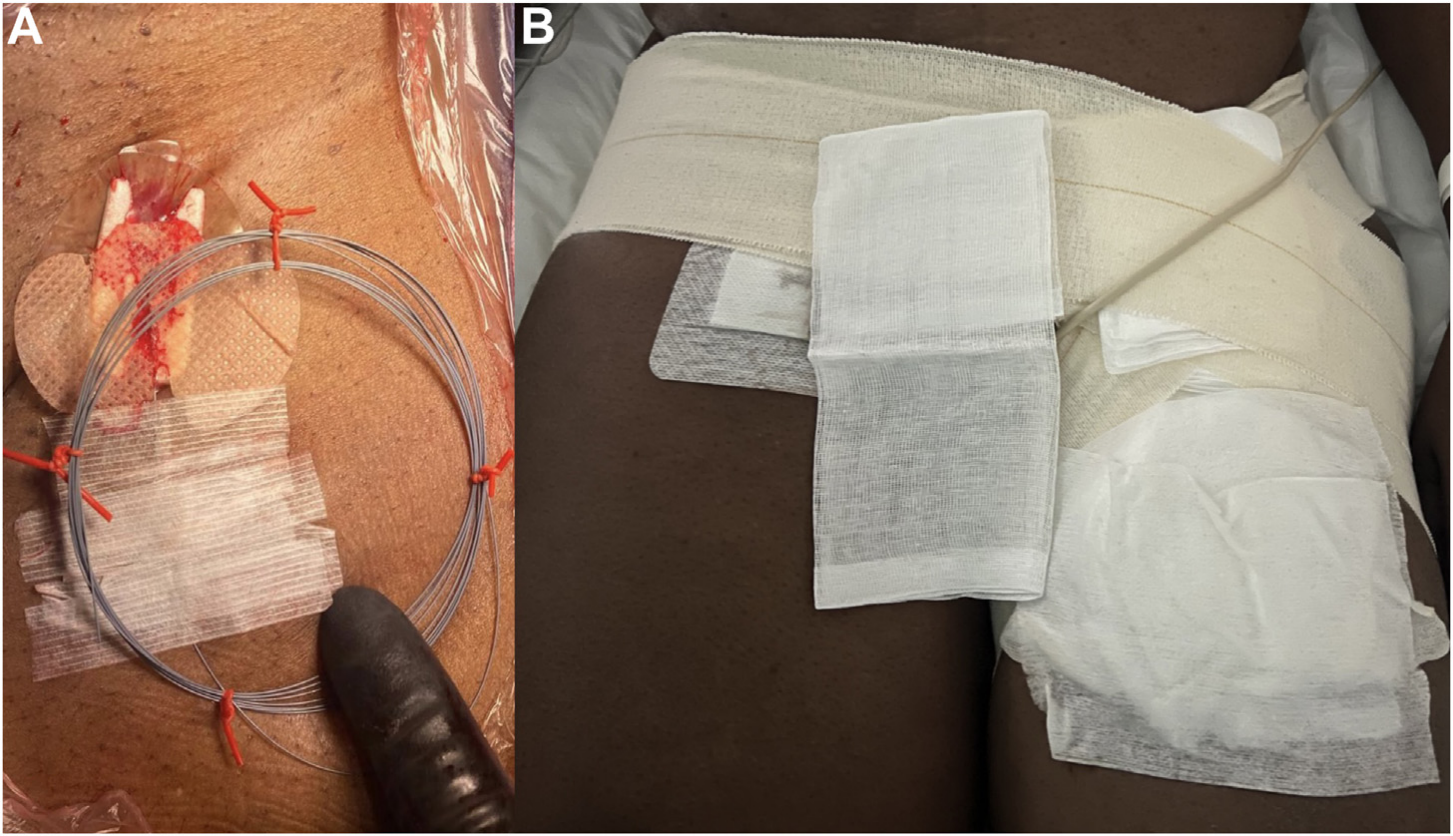
Detail of postoperative management of femoral artery access. After percutaneous closure, the V-18 guidewire is rolled-up **(A)** and draped in sterile fashion with a gentle compressive bandage applied **(B)**.

In both cases, at the end of the endovascular procedure, the V-18 guidewire was wrapped in a sterile fashion and the patient monitored in the intensive care unit for 48 hours (Fig 4), with a continuous unfractionated heparin infusion and a target activated clotting time of 160 to 200 seconds. Aspirin was reintroduced 24 hours after the procedure, and no episodes of bleeding around the wire exit site were reported. After 48 hours with an uneventful course, the guidewire was simply pulled out from the groin at the patient’s bedside, with no need for groin compression and no related complications occurring.

### Protocol in case of SCI

In the case of SCI, the patient is rapidly moved to the operating room. Under local anesthesia, a 6F introducer sheath is advanced over the 0.018-in. guidewire. A 5F Berenstein catheter is advanced through the 0.018-in. guidewire in a trans-sealing fashion into the aneurysmal sac, and the V-18 guidewire is exchanged for a 0.035-in. stiff guidewire. A 6F, 90-cm Destination sheath (Terumo) is then advanced trans-sealing over the stiff guidewire in the aneurysmal sac. If the Destination sheath does not advance, a low-profile, 6-mm-diameter balloon can be advanced trans-sealing over the 0.018-in. guidewire and gently inflated, and the Destination sheath can be pushed over the balloon while the balloon is slowly and progressively deflated.

**Fig 5:**
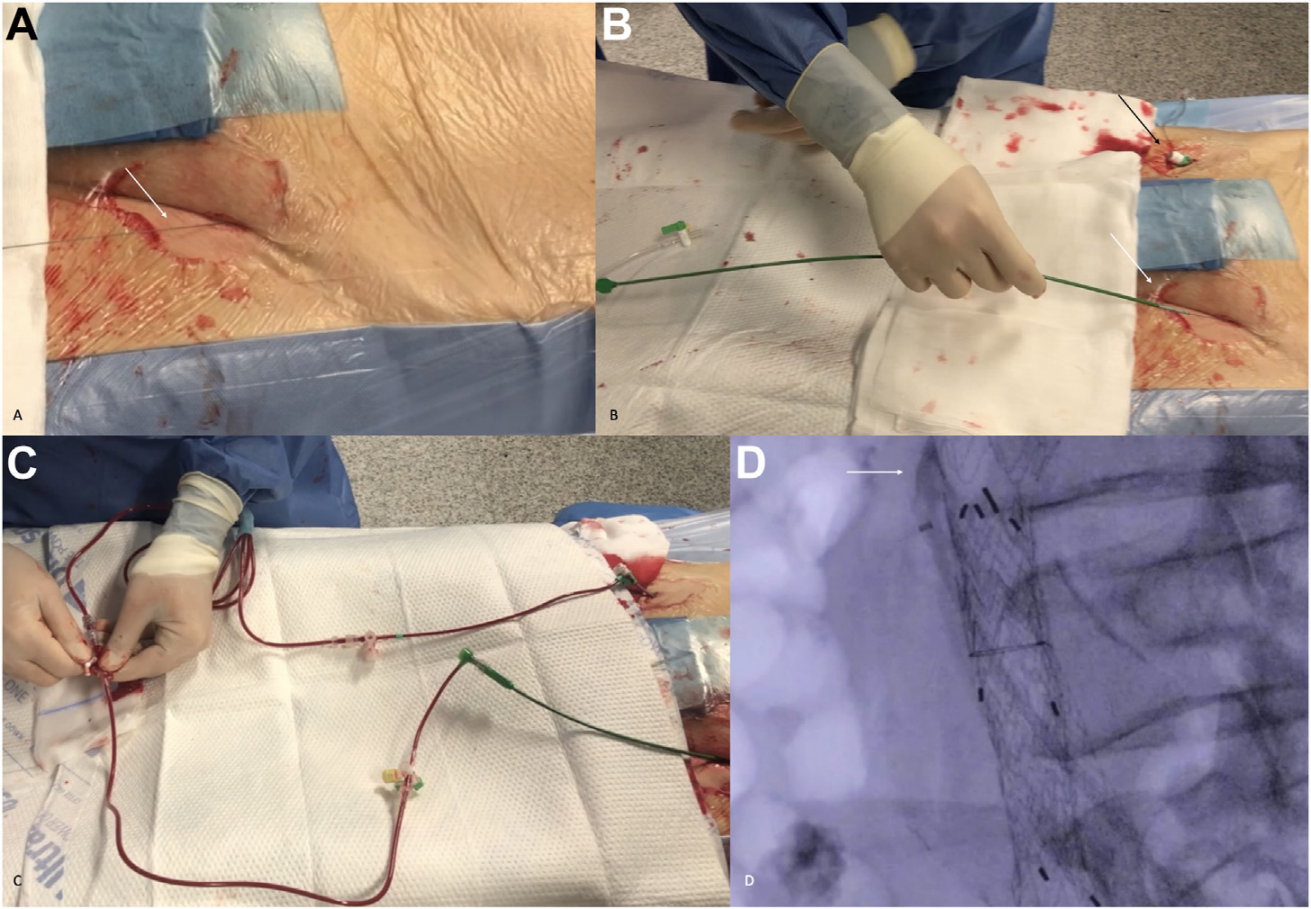
Use of the “safe line” in the case of spinal cord ischemia (SCI). The guidewire is unwound **(A)**, a 6F Destination introducer sheath is inserted directly into the aneurysm sac **(B)** and connected to the contralateral femoral percutaneous access via a three-way runner, reperfusing the aneurysmal sac **(C)**. **D,** Intraoperative angiogram from the Destination sheath, confirming its correct position in the aneurysm sac (*white arrow*) with sac reperfusion.

At this point, a 6F introducer sheath can be placed under local anesthesia into the contralateral common femoral artery and connected to the 6F Destination sheath through a three-way stopcock, which will also allow for continuous pressure measurements with a transducer through the “T” line (Fig 5). The flow load and modality for sac perfusion can be at the operator’s discretion (eg, creating a type Ib leak with a balloon, increasing the size to a 7F or 8F system, use of an external pump), balancing the benefits of symptom relief with the risk of complications related to the high-velocity jet into the sac.

The system can be left in place for a variable number of hours and the cerebrospinal fluid (CSF) drain placed, maintaining a target pressure of <10 mm Hg. Also, a mean arterial pressure of >90 mm Hg and hemoglobin level of >10 mg/dL should be maintained. The subsequent management will depend on the clinical evolution of the SCI symptoms. The system can be removed at the patient’s bedside in the intensive care unit, applying manual compression to the access site and an elastic bandage. A percutaneous closure system can also be used.

## Discussion

Surgical strategies to prevent SCI are based on staging the procedure through multiple steps of aortic coverage, TASP, or minimally invasive segmental artery coil embolization.^5^^,^^7^, ^8^, ^9^, ^10^ In 2018, Gombert et al^11^ reported an in vitro study on the use of a slowly occluding hydrogel-textile membrane that could be useful in the future.

However, once endovascular exclusion has been completed, none of these techniques allow for spinal cord reperfusion, and the only measures that can be used for SCI mitigation consist of spinal drainage and medical management (blood pressure and hemoglobin level optimization).

One report described a dedicated branch for sac temporary perfusion based on delivering, as a final endovascular step, an Amplatzer plug into the branch for ∼4 hours, with complete detachment after this period if the patient has no signs of SCI.^12^ However, maintenance of an Amplatzer plug in position for such a short period (4 hours) is a limitation because >65% of SCI cases can occur 24 hours after completion.^13^ Also, the heparin infusion through the sheath side branch can prevent complete thrombosis of the plug and affect sac exclusion. Furthermore, all methods based on temporary sac perfusion carry the limitation that the large “intentional” leak could increase the risk of rupture and disseminated intravascular coagulopathy during the waiting time before the final step.

To the best of our knowledge, the “safe-line” method is the first reported technique that can eventually be used as an ultimate attempt for fast sac reperfusion in the case of SCI occurring ≤48 to 72 hours after complete exclusion. This represents a simple and fast procedure that does not increase the complexity of surgery or the procedural time and, if needed, can guarantee fast sac reperfusion after symptom occurrence. To reduce the theoretical risk of iliac–femoral axis thrombosis in the early postoperative period, only the 0.018-in. guidewire is left in place, without any introducer sheath, via standard percutaneous access.

Furthermore, this technique does not require modification of the other standard SCI prevention methods. Also, in our protocol, it is used just as an adjunct to TASP and CSF drainage. If a spinal drain must be performed postoperatively because of SCI occurrence,^12^ the heparin infusion can be stopped and the “safe-line” can be rapidly (within 1 hour) activated. During this time, the coagulation parameters can be adequately restored to an activated clotting time of <150 seconds. The continuous pulsatile flow in the circuit should guarantee a low risk of thrombosis, and the CSF drainage tube can be safely inserted.^14^

If no clinical benefit is realized within 4 to 6 hours, we believe the system can be removed. If clinical improvement in SCI has occurred within 1 to 3 hours, the system can be maintained, with a low dose of heparin reintroduced 6 hours after drainage insertion. Within the limitations of our initial experience, we believe this technique can be applied to patients with elective type I, II, or III TAAAs undergoing the final step or treated in a single stage and all patients with urgent type I, II, or III TAAAs without frank rupture. Regarding the anatomic factors, the “safe-line” might be less useful in cases of extensive posterior chronic mural thrombus with no evidence of intercostal arteries on the preoperative computed tomography angiogram. However, for patients with patent large and multiple intercostal arteries, this technique can be considered. For postdissection TAAAs, we think that the “safe-line” role must be carefully evaluated. Placing the guidewire in the correct lumen from which the major collateral vessels arise could be cumbersome. Also, the pulsatile flow in a dissected area could result in a greater risk of sac complications. Regarding the level of the catheter into the sac, we believe it should be positioned in an area (abdominal infrarenal, paravisceral, or distal thoracic) where the major intercostal arteries have been identified on the preoperative computed tomography angiogram. If needed to evaluate SCI, an aneurysm sac angiogram can be performed to evaluate changes in the perfusion of the collateral vessels, with positioning of the catheter at the level of the remaining patent collateral arteries.

The limits of the described technique are primary that the safety and efficacy require further evaluation in additional and larger multicenter studies.

## Conclusions

The safe-line technique consists of maintaining guidewire access to the aneurysm sac for rapid reperfusion in the case of the development of SCI after complete endovascular reperfusion. This approach appears to be feasible; however, further data are necessary to establish its efficacy and role in SCI prevention.

## Appendix (online only)

Supplementary Video (online only) Video detailing the rational and procedural steps of the safe-line technique.
